# Coactosin Promotes F-Actin Protrusion in Growth Cones Under Cofilin-Related Signaling Pathway

**DOI:** 10.3389/fcell.2021.660349

**Published:** 2021-06-21

**Authors:** Xubin Hou, Motohiro Nozumi, Harukazu Nakamura, Michihiro Igarashi, Sayaka Sugiyama

**Affiliations:** ^1^Laboratory of Neuronal Development, Graduate School of Medical and Dental Sciences, Niigata University, Niigata, Japan; ^2^Department of Molecular Neurobiology, Graduate School of Life Sciences, Institute of Development, Aging and Cancer, Tohoku University, Sendai, Japan; ^3^Department of Neurochemistry and Molecular Cell Biology, Graduate School of Medical and Dental Sciences, Niigata University, Niigata, Japan

**Keywords:** actin cytoskeleton, axon outgrowth, COTL1, growth cone, structured illumination microscopy

## Abstract

During brain development, axon outgrowth and its subsequent pathfinding are reliant on a highly motile growth cone located at the tip of the axon. Actin polymerization that is regulated by actin-depolymerizing factors homology (ADF-H) domain-containing family drives the formation of lamellipodia and filopodia at the leading edge of growth cones for axon guidance. However, the precise localization and function of ADF-H domain-containing proteins involved in axon extension and retraction remain unclear. We have previously shown that transcripts and proteins of coactosin-like protein 1 (COTL1), an ADF-H domain-containing protein, are observed in neurites and axons in chick embryos. Coactosin overexpression analysis revealed that this protein was localized to axonal growth cones and involved in axon extension in the midbrain. We further examined the specific distribution of coactosin and cofilin within the growth cone using superresolution microscopy, structured illumination microscopy, which overcomes the optical diffraction limitation and is suitable to the analysis of cellular dynamic movements. We found that coactosin was tightly associated with F-actin bundles at the growth cones and that coactosin overexpression promoted the expansion of lamellipodia and extension of growth cones. Coactosin knockdown in oculomotor neurons resulted in an increase in the levels of the inactive, phosphorylated form of cofilin and dysregulation of actin polymerization and axonal elongation, which suggests that coactosin promoted axonal growth in a cofilin-dependent manner. Indeed, the application of a dominant-negative form of LIMK1, a downstream effector of GTPases, reversed the effect of coactosin knockdown on axonal growth by enhancing cofilin activity. Combined, our results indicate that coactosin functions promote the assembly of protrusive actin filament arrays at the leading edge for growth cone motility.

## Introduction

During embryonic brain development, neuronal growth cones are identified by the presence of large lamellipodia with sharp filopodia projecting from the growing edge of axons. The structural features are largely derived from the regulation of actin arrays (Welnhofer et al., 1997; Dent et al., 2011; Gomez and Letourneau, 2014), which indicates that actin dynamics can operate the motility of growth cones. Many actin-binding proteins are found in the growth cone (Ishikawa and Kohama, 2007; Nozumi et al., 2009), which suggests that these molecules are involved in axon formation and development.

Actin-depolymerizing factor homology (ADF-H) domain-containing proteins are essential for actin treadmilling and polymerization/depolymerization processes in which actin monomers are removed from the pointed end and added to the barbed end of actin filaments (Lappalainen et al., 1998; Yang et al., 1998; Poukkula et al., 2011). For instance, ADF/cofilin, an extensively characterized F-actin severing protein, more strongly binds to ADP-actin than do ATP- and ADP-Pi actin filaments and accelerates depolymerization by removing actin monomers from the pointed ends of actin filaments (Yang et al., 1998; Bamburg, 1999, Kiuchi et al., 2007). Consequently, the F-actin severing activity of ADF/cofilin allows for a spatially targeted disassembly that can decrease F-actin length and promote actin turnover at the lamellipodia (Bamburg and Bray, 1987; Bamburg, 1999; Schaefer et al., 2008; Flynn et al., 2012). In contrast, drebrin and coactosin bind only to F-actin, and not to actin monomers (Lappalainen et al., 1998; Provost et al., 2001; Esser et al., 2009). Importantly, coactosin prevents cofilin-mediated depolymerization, thereby promoting lamellipodia formation (de Hostos et al., 1993; Röhrig et al., 1995; Provost et al., 2001; Hou et al., 2013; Kim et al., 2014). Despite the increasing number of biochemical and structural studies relating to ADF-H family proteins, their roles in regulating actin dynamics in cellular structures are not yet incompletely understood.

Coactosin is colocalized with actin stress fibers in mammalian cells (Provost et al., 2001; Tojkander et al., 2012) and the migration of cultured neural crest cells (Hou et al., 2009, 2013). Knock-down of coactosin disrupts actin polymerization in actin stress fibers, whereas its overexpression increases the formation of cellular protrusions (Hou et al., 2013). Moreover, coactosin is recruited to protrusions of lamellipodia and filopodia in response to Rac signaling, as evidenced by the fact that a mutant form of coactosin that cannot bind to F-actin does not respond to Rac signaling and does not support cell migration (Hou et al., 2013).

Considering that coactosin is localized to the tips of cellular protrusions (Hou et al., 2013), this ADF-H domain-containing protein may be associated with actin filament arrays at the leading edge of growth cones and regulate their motility. In the present study, we found that coactosin was involved in growth cone through binding to actin filaments both *in vitro* and *in vivo*. A superresolution microscopic device, structured illumination microscopy (SIM) revealed that coactosin was localized to a distal part of actin bundles at the leading edge of growth cones. Moreover, coactosin knockdown inhibited axonal growth, concomitant with an increase in the levels of the inactive, phosphorylated form of cofilin. Finally, coactosin knockdown could restore the disoriented axon growth induced by LIMK1 inhibition through optimizing cofilin activity. Taken together, our results demonstrated that coactosin was associated with actin bundles and, through optimizing cofilin activity, promoted axon outgrowth via actin remodeling.

## Materials and Methods

### Chick Embryos

Fertile chicken eggs were obtained from a local supplier (Yamagishi, Japan) and incubated at 38°C in a humid atmosphere until the desired stages (Hamburger and Hamilton, 1951).

### Expression Vectors

Hemagglutinin (HA)-tagged coactosin cDNA was cloned into the pMiw expression vector (containing the Rous sarcoma virus enhancer and the beta-actin promoter) (Suemori et al., 1990; Wakamatsu and Weston, 1997; Sugiyama et al., 2000) and the pCAGGS expression vector [containing the cytomegalovirus (CMV) enhancer and the chicken beta-actin promoter] (Niwa et al., 1991). To generate EGFP fusion protein, coactosin (GenBank accession number AB519794) was separately inserted into pEGFP-C1 (Clontech/Takara Bio, Japan). To generate mutant coactosin, lysine75 (actin-binding site) was changed to alanine using a mutagenesis kit from (Stratagene/Agilent Technologies, United States) and inserted into pMiw and phrGFP (Clontech). Dominant–negative forms of RhoA (GenBank accession number M27278) and LIMK1 (GenBank accession number AB073752) were inserted into the pEF-BOS-HA × 3 vector (a kind gift from Dr. Kazumasa Ohashi). For visualizing LacZ or GFP in axons, tau-LacZ or tau-GFP cDNA was cloned into the pMiw expression vector.

### Short Hairpin RNA Construction

Short hairpin RNA (ShRNAs) targeting four distinct regions within the coactosin coding sequence were tested (Katahira and Nakamura, 2003; Hou et al., 2011). ShRNA-Coactosin350 (5′-ACAAAGAGCTGGATGAGGACTA-3′) was the most effective and was inserted into the shRNA expression vector pRFPRNAiA (a kind gift from Dr. Stuart Wilson) containing the chick U6 promotor and a miRNA-like hairpin insertion site (TAGTGAAGCCACAGATGTA), which allows for the simultaneous expression of the shRNA and RFP. To co-express the shRNA and GFP, shRNA-Coactosin350 was inserted into pSuper-GFP (Dr. Matsumoto. K., license obtained from Clontech), which contains the H1 promoter and a nine-base hairpin loop sequence (5′-TTCAAGAGA-3′) (Hou et al., 2013). A construct containing scrambled shRNA was used as a control. We confirmed that these shRNAs do not cross-react with chick destrin or cofilin using BLAST search.

### In Ovo Electroporation

In ovo electroporation was performed as previously described (Funahashi et al., 1999). Briefly, a solution containing 1 μg/μL plasmid was injected into the lumen of the chick neural tube and the central canal at stages 10–12 and at stage 14, respectively. The electrodes were placed on the vitelline membrane next to the brain vesicles, and 4 × 25 V, 50 ms rectangular pulses were applied by an electroporator (CUY21 edit, BEX). Only the anode side was transfected, with the untransfected side serving as the control.

### Primary Culture of Oculomotor Neurons

After washing in nitric acid and sterilizing, glass coverslips were coated with 10 mg/mL poly-L-lysine (Sigma-Aldrich, United States). The ventral mesencephalon was dissected from stage 13–14 chick embryos, quickly washed in Ca^2+^/Mg^2+^-free (CMF) Hanks balanced salt solution (HBSS, Gibco/Thermo Fisher Scientific, United States), treated with 0.25% trypsin in CMF HBSS for 30 min at 37°C, and then with DNase for a further 2–3 min. The tissue fragments were then washed in warm CMF HBSS and triturated in CMF HBSS to yield a single-cell suspension. Cells were cultured in Dulbecco’s modified Eagle’s medium (DMEM)/F-12 supplemented with 2% B27, 10 mM 4-(2-hydroxyethyl)-1piperazine ethane sulfonic acid (HEPES, DojinDo, Japan), and 1–10 ng/mL nerve growth factor (NGF, Invitrogen/Thermo Fisher Scientific, United States) for 24 h.

For live-cell imaging and immunostaining, expression vectors or shRNAs were introduced into cells by electroporation (CUY21 edit, BEX). After electroporation, the cells were replated on a glass coverslip or glass-bottom dish precoated with 0.05% polyethylenimine (PEI).

For the cell death assay, the *In Situ* Cell Death Detection Kit, TMR red (Roche Applied Science, Switzerland) was employed according to the manufacturer’s protocol. The electroporation of vectors for gain- and loss-of-function analysis did not result in cell death as determined by a TUNEL assay.

### Cell Lines

NG108-15 was cultured in DMEM (WAKO, Japan) supplemented with 10% fetal bovine serum (Nozumi et al., 2009). Following electroporation of the expression vectors (CUY21 edit, BEX), the cells were replated, and the medium was replaced with Leibovitz’s L-15 medium (Gibco) to induce the differentiation of NG108-15 cells. Growth cones were induced from 3 h after serum starvation.

### Live-Cell SIM Imaging

Structured illumination microscopy images were acquired using an open heating chamber attached to an inverted microscope (Zeiss ELYRA S.1) equipped with a Plan Apochromat oil immersion lens (63×, 1.4 NA) (Nozumi et al., 2017; Igarashi et al., 2018). Image acquisition, SIM processing, and channel alignment were performed using the Zeiss software ZEN 2011 with reference to TetraSpeck fluorescent bead standards (0.1 μm, Invitrogen).

To analyze the rate of growth cone extension, the geometric center of growth cones was defined from the leading edge in the movies (70 frames/700 s), and the distance traveled of growth cones was quantitatively analyzed by tracking the geometric center using MTrackJ, which is an ImageJ plugin (Fiji software) to facilitate tracking. The area of lamellipodia was measured as the region of interest using an enhanced phalloidin staining image, and the area changes were calculated from the movies (70 frames/700 s).

### *In situ* Hybridization and Immunochemistry

Whole-mount and section *in situ* hybridization were performed as previously described (Hou et al., 2013). Chick *Cotl1* subclones (Hou et al., 2009) were linearized and digoxigenin (DIG)-labeled antisense RNA was generated (Stratagene).

The following primary antibodies were used for immunohistochemistry: anti-Coactosin (Sawady Technology, Tokyo, Japan), which was raised in rabbits using the bacterially expressed peptide NH2-DHKELDEDYIKNELK-COOH as previously described (Hou et al., 2009); anti-HuC/D (Molecular Probe); anti-neurofilament (3A10; Developmental Studies Hybridoma Bank [DSHB]), anti-Islet1/2 (2D6; DSHB); anti-cofilin (Sigma); anti-*p*-cofilin (Ser3) (sc-21867-R; Santa Cruz Biotechnology); anti-HA (3F10; Roche Applied Science); and anti-GFP (Invitrogen). Alexa-conjugated anti-mouse, anti-rat, and anti-rabbit antibodies were used as secondary antibodies (Invitrogen).

After SIM imaging, immunostaining was carried out as previously described (Nozumi et al., 2009). Cells were fixed in 1% glutaraldehyde in PBS (137 mM NaCl, 2.7 mM Na_2_HPO_4_, 8.1 mM KCl, and 1.5 mM KH_2_PO_4_), permeabilized with 0.1% Triton X-100 in PBS, and reacted with primary and secondary antibodies. Rhodamine-phalloidin was diluted in the secondary antibody solution. For glutaraldehyde fixation, background staining was reduced by treatment with 1% sodium tetrahydroborate.

### Imaging Analysis

For analysis of SIM imaging, fluorescent intensities of anti-coactosin, anti-cofilin, anti-*p*-cofilin Abs, and rhodamine-phalloidin were acquired as the gray value per pixel to a selected staining area of a given growth cone (Nozumi et al., 2017; Igarashi et al., 2018). The value of intensity (per pixel) was normalized as a ratio to the maximum value of intensity, and values greater than 0 a.u. was used as positive signals. The intensity in the region of interest was measured using the Zen software (Zeiss), and the number of fluorescent puncta was counted using the Analyze Particles mode of the ImageJ software (user guide 30.2^1^). The distances from the leading edge were calculated using the outline of a growth cone that was distinguished from the background fluorescence signals. The fluorescence signals in the “between bundles” groups were defined as the appearance of the signal of coactosin or cofilin between non-crossing actin bundles. We also defined the fluorescence signals in the “on bundle” when the signal overlapped with a single actin bundle. All statistical values obtained from experiments are presented as means ± standard error of the mean (SEM).

## Results

### Coactosin Localized to the Growth Cones of Tectobulbar Axons in the Midbrain

**FIGURE 1:**
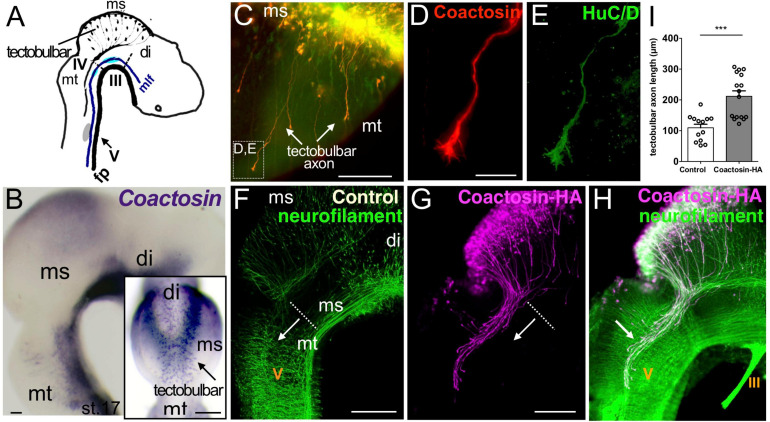
Coactosin localized to growth cones of tectobulbar axons of the midbrain. **(A)** Schematic representation of axonal tracts in the chick mesencephalon (ms). Tectobulbar neurons project their axons ventrally, turn caudally before reaching the tegmentum, and elongate alongside medial longitudinal fascicule (mlf). **(B)** Expression of coactosin mRNA in the dorsal midbrain (tectum) in chick embryos at stage 17. Dorsal view (inset) showing the strong spot-like expression around the dorsal midline (arrow, tectobulbar neuron). **(C–E)** Coactosin (*red*) and HuC/D (*green*) proteins within tectobulbar axons in the mid-hindbrain region. Tectobulber axons project ventrally from stage 16 (arrows). Higher magnification showing coactosin and HuC/D distribution in the growth cones **(D,E**; indicated in **C)**. **(F–H)** The overexpression of hemagglutinin (HA)-tagged coactosin leads to the premature elongation of tectobulbar axons at the electroporation side (arrow; **G,H**) when compared with the control side (arrow; **F**). Double staining with the HA-tag (*magenta*) and neurofilament (*green*) antibodies again shows the axonal distribution of HA-tagged coactosin **(H)**. **(I)** Tectobulbar tracts showing axonal elongation with coactosin overexpression, compared with the control (13 and 16 embryos, ***p = 0.0001, Mann–Whitney *U* test). The length of the axons was measured from the mid-hindbrain boundary (dot-line). Abbreviations: mt, metencephalon; di, diencephalon; III, oculomotor nucleus; IV, trochlear nucleus; V, trigeminal ganglion. Scale bar, 200 μm in panel **(B,F,G)**; 50 μm in panel **(C,D)**.

We previously found that coactosin is an ADF-H domain-containing protein that promotes actin remodeling in migrating neural crest cells in the chick embryo (Hou et al., 2013). In that study, we further showed that coactosin mRNA was expressed in the mesencephalon during neural development. Here, we found that coactosin was strongly expressed in a spot-like pattern near the dorsal midline of the mesencephalon from stage 17 (Figures 1A,B). In the dorsal midline, Coactosin-expressing cells were early differentiated among mesencephalic cells and expressed the neural marker HuC/D (Figures 1C–E). These early-differentiated neurons extended neurites, which were recognized as tectobulbar axons. Coactosin was evidently incorporated in the tectobulbar axons. A higher magnification revealed the distribution of coactosin in the lamellipodium of the growth cone (Figures 1D,E).

To investigate the role of coactosin in axons, we overexpressed HA-tagged coactosin in cells extending tectobulbar axons using *in ovo* electroporation. Coactosin was overexpressed in only one side of each embryo, with the unelectroporated side serving as a control. At stage 20 of embryonic development, the tectobulbar axons on the control side stayed in the mesencephalon (Figure 1F). In contrast, coactosin-overexpressing neurons prematurely extended tectobulbar axons to the metencephalon and the axons entered the tectobulbar tract by coursing caudally within the metencephalon (Figures 1G–H, arrow). The length of coactosin-overexpressing tracts was significantly elongated from the midbrain-hindbrain boundary into the metencephalon compared with the control side, which was detected by neurofilament immunostaining at the unelectroporated side in the same embryo and/or GFP expression after electroporation (Figure 1I; control, 109.4 ± 11.7 μm vs. coactosin, 211.7 ± 17.7 μm; 13 and 16 embryos, *p* = 0.002, Mann–Whitney *U* test). This suggested that coactosin in the growth cone was involved in axonal extension.

### Coactosin Knockdown Impaired Axonal Elongation in the Embryonic Oculomotor Nerve

**FIGURE 2:**
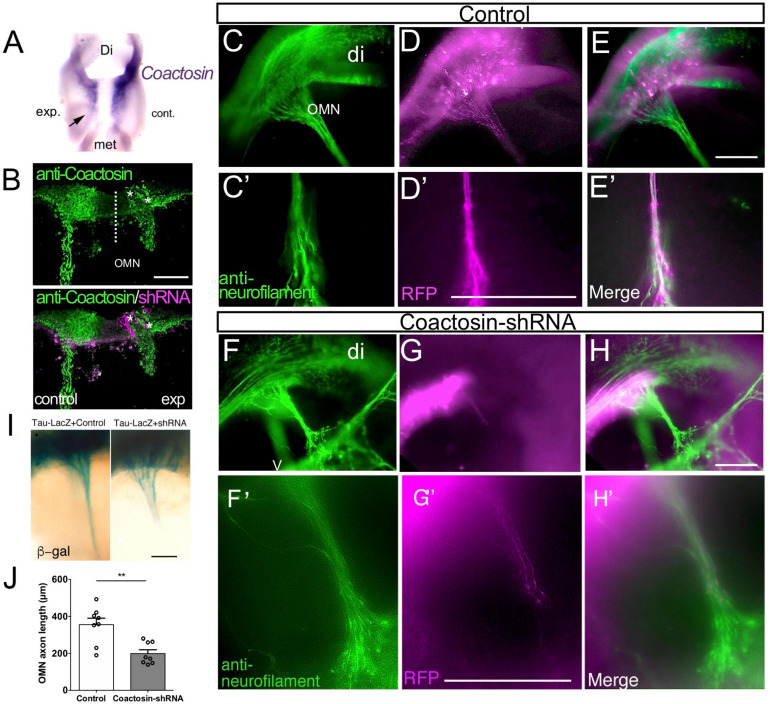
Effects of coactosin knockdown on elongation of the oculomotor nerve. **(A)** Reduction in coactosin mRNA levels following transfection with coactosin short hairpin RNA (shRNA). Ventral view of the midbrain showing the weak expression on the experimental side (exp) compared with that in the control side (cont). **(B)** Coactosin (*green*) expression is reduced in the coactosin shRNA-/RFP-expressing oculomotor nucleus (OMN) on the experimental side (asterisks). **(C–H)** Effects of scrambled (control) shRNA **(C–E)** or coactosin shRNA (**F–H**) transfection on the oculomotor nerve. Double staining of RFP and neurofilament (*green*) shows the control shRNA-transfected axons extending toward an eye (**C′–E′:** higher magnification of **C–E**). In contrast, coactosin shRNA transfection disrupted the elongation of the oculomotor nerve (**F′–H′**: higher magnification of **F–H**). **(I)** Effects of control shRNA (left) or coactosin shRNA (right) transfection on the tau-LacZ-expressing oculomotor nerve. The tau-lacZ-labeled axons paused at the midway of their migration route following coactosin knockdown. **(J)** The length of oculomotor axons from its nucleus shows a shrinkage with coactosin shRNA than control shRNA (eight embryos for each, **p = 0.0047, Mann–Whitney *U* test). Scale bar: 100 μm.

To investigate the function of coactosin in axonal extension, coactosin was knocked down in stage 14 embryos by electroporation of the pRFPRNAiA expression vector, which allowed the simultaneous expression of the shRNA targeting coactosin and RFP. We confirmed that, compared with the control side, the expressions levels of endogenous coactosin mRNA and protein were both markedly reduced in the oculomotor nucleus at the coactosin shRNA-expressing side (Figures 2A,B). After the electroporation of the scrambled shRNA (control) vector, RFP-positive axons were assembled into a long and broad neurofilament-positive nerve bundle (oculomotor nerve) and extended from the ventral mesencephalon toward the eyeball at stage 20 (Figures 2C–E; Chilton and Guthrie, 2004). In contrast, RFP-positive oculomotor axons were shorter with coactosin knockdown, and these axons were paused midway along their migration route, unlike RFP-negative axons (Figures 2F–H) or RFP-positive axons expressing the control shRNA (Figures 2C–E). Oculomotor axons that extended from the nucleus had a significantly shorter length with coactosin shRNA than with control shRNA (Figure 2J; control shRNA, 358.5 ± 37.6 μm vs. coactosin shRNA, 210.4 ± 22.3 μm, eight embryos for each, *p* = 0.0047, Mann–Whitney *U* test). Similar results were obtained when shRNA was co-expressed with the tau-LacZ protein in which tau could transport LacZ protein to the axon tips (Figure 2I). These results indicate that coactosin knockdown impaired oculomotor nerve elongation.

### The Precise Association Between Coactosin and F-Actin Bundles at the Leading Edge of the Growth Cone

The growth cone of a NG108-15 cell is morphologically characterized by large, flattened lamellipodia with sharp filopodia, and thus is an ideal system for observing actin arrays (Nozumi et al., 2017). To visualize the association between coactosin and actin bundles in detail, we obtained time-lapse images of NG108-15 cells using SIM (Movies), which is the most suitable superresolution microscopic device with which analyze the dynamics of cellular components (Hirano et al., 2015; Heintzmann and Huser, 2017; Igarashi et al., 2018; Schermelleh et al., 2019).

**FIGURE 3:**
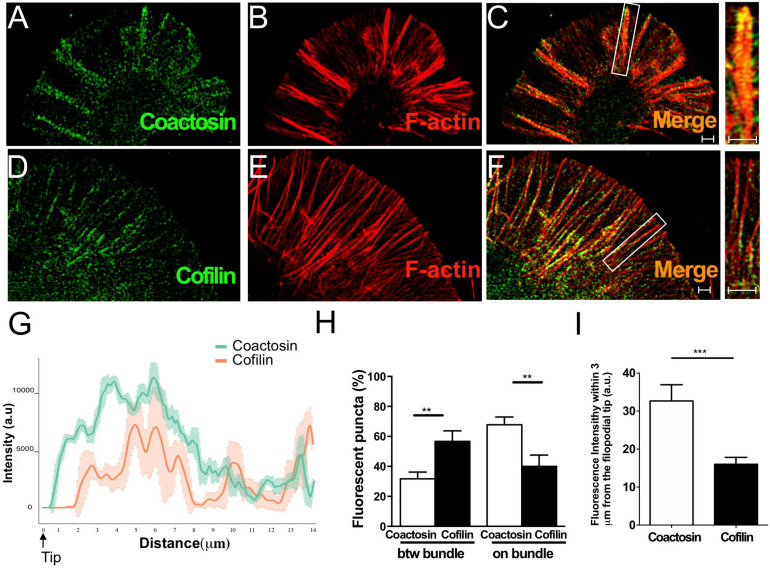
Distribution of coactosin and cofilin in the growth cones of NG108-15 cells. **(A–C)** SIM images showing the co-localization of endogenous coactosin (*green*) and F-actin (rhodamine-phalloidin, *red*) in a growth cone. **(D–F)** Co-localization of cofilin (*green*) and F-actin (*red*) in a growth cone. Note that cofilin accumulation on F-actin is weak at the tip of filopodia (inset). **(G)** Quantitative distribution of the fluorescence intensities of coactosin (*green*) and cofilin (*red*). Measurements were performed 14 μm from a filopodial tip (*n* = 18 for each, means ± SEM). **(H)** Quantitative analysis of the association of coactosin or cofilin with F-actin. Approximately 68% of the coactosin-positive pixels were located on actin bundles (“on bundle”), while the rest were located between actin bundles (“btw bundle”). The data are shown as means ± SEM (19–26 bundles, **p < 0.01, Mann–Whitney *U*-test). **(I)** Quantification of the percent association with F-actin within 3 μm of the filopodial tip. The data are shown as means ± SEM (21–24 bundles, ***p = 0.0005, Mann–Whitney *U*-test). Scale bars, 1 μm.

We previously reported that coactosin is located at the tip of filopodia of neural crest cells and induces filopodial extension (Hou et al., 2013). As expected, we observed that intrinsic coactosin was localized to filopodia that contained the bundled F-actin, and was partially distributed to lamellipodia between the bundles at the peripheral (P)-domain of growth cones (Figures 3A–C). In contrast, cofilin was discontinuously distributed along the actin bundles and sparsely expressed at the distal part of the growth cone (Figures 3D–F). Quantitative analysis of the fluorescence intensities in the filopodia showed that coactosin, and not cofilin, continuously accumulated near the tips of filopodia (Figure 3G). Coactosin distribution was quantified by measuring coactosin-positive pixels relative to phalloidin-positive pixels at the P-domain of growth cones. Approximately 68% of coactosin-positive puncta were distributed on phalloidin-positive actin bundles within the filopodia while the remaining puncta were located between the bundles (lamellipodia) (Figure 3H). In addition, compared with the sparce distribution of cofilin, coactosin was abundantly detected even within 3 μm from the tip of filopodia (Figure 3I). SIM imaging results revealed the precise association between intrinsic coactosin and actin filaments.

### The Effect of Coactosin Association With F-Actin Bundles in the Highly Motile Growth Cone

**FIGURE 4:**
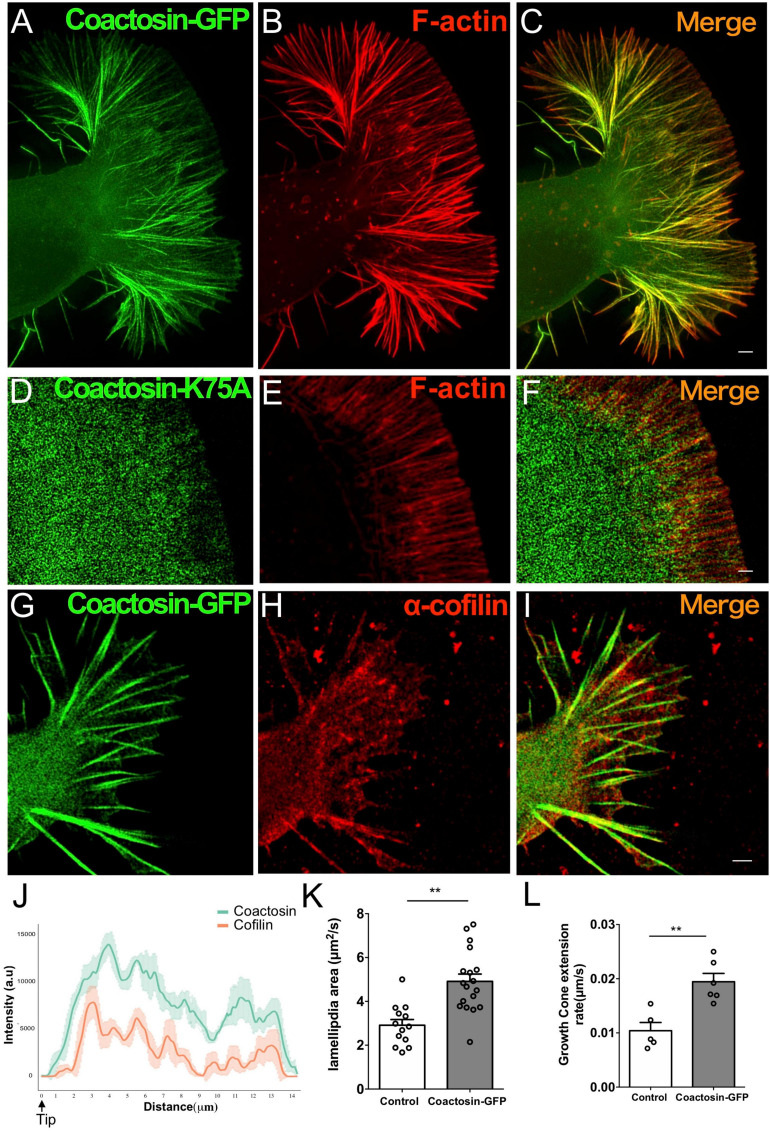
Association of coactosin with F-actin extends the growth cones of NG108-15 cells. **(A–C)** Co-localization of GFP-coactosin (*green*) and F-actin (rhodamine-phalloidin, *red*) in SIM images of a growth cone. **(D–F)** Distribution of mutated coactosin (K75A) in a growth cone. Coactosin K75A (*green*) that cannot bind F-actin did not accumulate on F-actin (*red*). **(G–I)** Distribution of cofilin with coactosin overexpression in a growth cone. Note that cofilin accumulation is weak at the tip of filopodia. **(J)** Quantitative distribution of the fluorescence intensities of GFP-coactosin (*green*) and cofilin (*red*) (*n* = 22 for each, means ± SEM). **(K)** The area change of lamellipodia shows rapid expansion with coactosin overexpression than control (also see Movies, 13–18 lamellipodium, **p = 0.0025, Mann–Whitney *U* test). **(L)** Increased rate of growth cone extension with coactosin overexpression compared with the control (5–6 cells, **p = 0.0031, Mann–Whitney *U* test). Scale bars, 1 μm.

Next, we observed the localization of introduced coactosin-GFP at the P-domain of growth cones of NG108-15 cells (Figures 4A–C; also see Supplementary Movie 1). Coactosin-GFP was localized at the actin bundles within the filopodia (Figure 4J) and further accumulated on the actin bundles than intrinsic coactosin (for “between bundles”, coactosin-GFP, 16.31 ± 2.41% vs. coactosin, 29.81 ± 4.36%, 23–26 bundles, *p* = 0.037, Mann–Whitney *U* test; for “on bundles”, coactosin-GFP, 81.77 ± 3.78% vs. intrinsic coactosin, 67.77 ± 5.14%, 19 bundles for each, *p* = 0.01, Mann–Whitney *U* test). Conversely, coactosin association with actin filaments was abolished when mutated coactosin (K75A) that cannot bind to actin was expressed (Provost et al., 2001; Hou et al., 2013) (Figures 4D–F). Moreover, coactosin overexpression did not influence the distribution of intrinsic cofilin, in which cofilin remained on the filopodial actin bundles except for at the tip of filopodia (Figures 4G–I,J).

To elucidate the effect of coactosin on actin polymerization, we examined the rate of lamellipodial expansion at the P-domain of growth cones. Quantitative analysis of the timelapse images showed that the area of lamellipodia was rapidly expanded with coactosin overexpression than with control GFP (Supplementary Movies 2, 3) and Figure 4K; 13 lamellipodia for control GFP and 18 lamellipodia for coactosin-GFP, *p* = 0.0025, Mann–Whitney *U* test). Moreover, the rate of growth cone extension was significantly higher with coactosin overexpression than with control GFP (Supplementary Movies 2, 3) and Figure 4L; 5 cells for control GFP and 6 cells for coactosin-GFP, *p* = 0.0031, Mann–Whitney *U* test). These results suggest that coactosin enhances actin polymerization at the leading edge of growth cones and consequently promotes the extension of growth cones.

### Endogenous Coactosin Colocalized With F-Actin Bundles in Axonal Growth Cones

**FIGURE 5:**
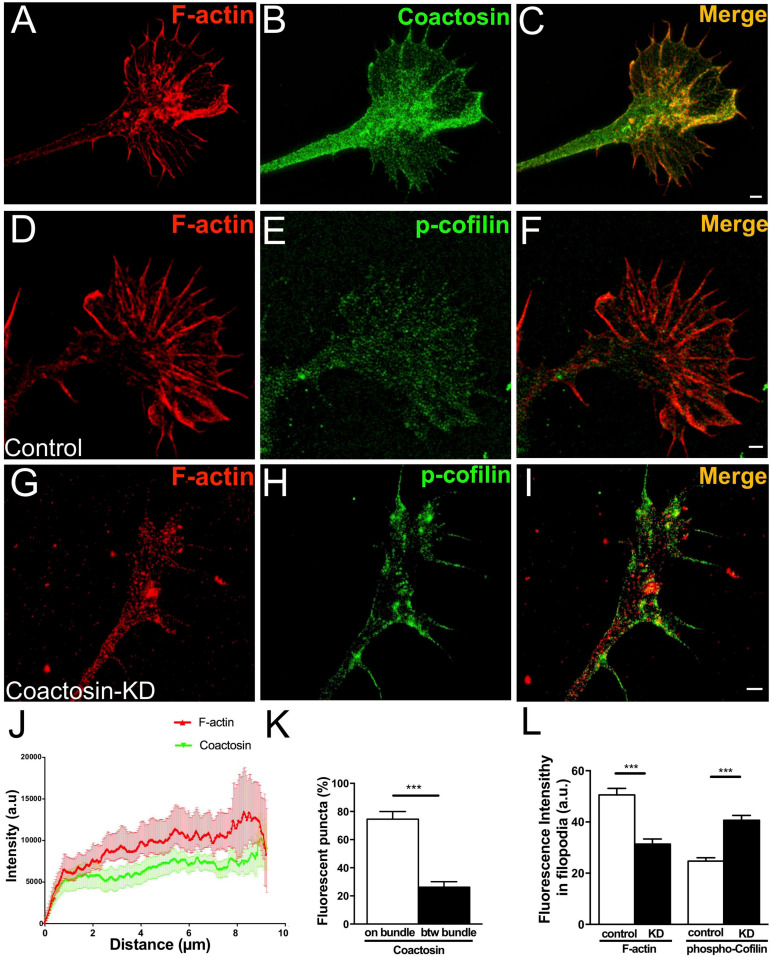
Concentration of coactosin signals needs at F-actin bundles. **(A–C)** Structured illumination microscopy (SIM) images showing endogenous coactosin and F-actin in the growth cone of an oculomotor neuron. Double staining of coactosin (*green*) and rhodamine-phalloidin (*red*) showing coactosin accumulation on F-actin bundles. **(D–I)** Representative SIM images of control **(D–F)** and coactosin-knockdown **(G–I)** neurons with double staining of phosphorylated cofilin (*p*-cofilin: *green*) and rhodamine-phalloidin (*red*). With coactosin knockdown, the actin array and bundle structures were disorganized and showed strong *p*-cofilin expression around the filopodia. **(J)** Quantitative distribution of the fluorescence intensities of coactosin (*green*) and F-actin (rhodamine-phalloidin, *red*). Measurements were performed 9 μm from a filopodial tip (*n* = 16 for coactosin and *n* = 24 for F-actin, means ± SEM). **(K)** Quantitative analysis of the association of coactosin or cofilin with F-actin. Approximately 75% of the coactosin-positive pixels were located on actin bundles (“on bundle”), while the rest of them were located between actin bundles (“btw bundle”). The data are shown as means ± SEM (25 bundles for each, ***p = 0.0001, Mann–Whitney *U*-test). **(L)** Quantitative fluorescence intensities of F-actin (rhodamine-phalloidin, *red*) and *p*-cofilin (*green*) in filopodia. Coactosin knockdown caused reduction of F-actin but accumulation of *p*-cofilin within filopodia (29 filopodia in 5 cells for each, ***p = 0.0002 for F-actin, *p* = 0.0005 for *p*-cofilin, Mann–Whitney *U*-test). Scale bars, 1 μm.

We then examined the precise localization of endogenous coactosin in the cultured oculomotor neurons using SIM. In the early phase of maturation, the neuronal growth cone, which is relatively smaller than the growth cone of NG108-15 cells, is characterized by the initial formation of a highly organized array of actin bundles. SIM imaging showed that coactosin protein was again localized to prominent F-actin foci in the growth cones of oculomotor neurons (Figures 5A–C). Notably, coactosin protein was preferentially localized to actin bundles within filopodia, as well as to radially-oriented actin filaments within the lamellipodia (between bundles) (Figures 5J,K).

To analyze whether coactosin influences cofilin activity in the growth cone, we knocked down coactosin expression by electroporation of a shRNA construct. Endogenous phosphorylated cofilin (the inactive form of cofilin) was sparsely and diffusely detected in the growth cones with control (scrambled) shRNA (Figures 5D–F). Conversely, phosphorylated cofilin was abundantly observed around the filopodia with coactosin shRNA (Figures 5G–I). Quantification analysis revealed that phosphorylated cofilin was significantly accumulated in the filopodia with coactosin knockdown compared with control (Figure 5L). Our previous study reported that the actin bundles are disrupted with coactosin knockdown in cultured neural crest cells (Hou et al., 2013). Similar to this observation, we found that coactosin knockdown resulted in the disruption of actin bundles in growth cones, particularly in the filopodia, whereas administration of control shRNA did not affect the array of actin bundles (Figures 5D,G,L). Our results suggest that coactosin is required for arrays of actin filaments in the growth cone *via* optimizing cofilin activity.

### Coactosin Was Involved in Rho GTPase-Mediated Axon Dynamics

**FIGURE 6:**
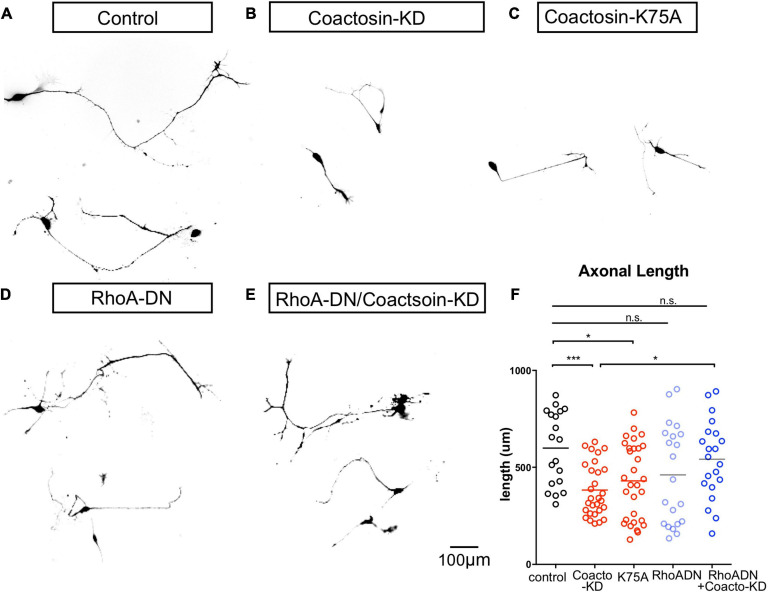
Coactosin function during axon growth under Rho signals. **(A–D)** Effects of coactosin on the elongation of cultured oculomotor neuron axons. Oculomotor neurons were transfected with control shRNA **(A)**, coactosin shRNA **(B)**, coactosin K75A **(C)**, a dominant-negative form of RhoA (RhoA-DN) **(D)**, or coactosin shRNA plus RhoA-DN **(E)**. **(F)** Quantification of axon length in oculomotor neurons. The data are shown as means ± SEM of three independent experiments (19–30 neurons, *p < 0.05; ***p < 0.0001, one-way ANOVA).

Our results indicated that coactosin was involved in preserving cofilin activity and F-actin arrays in the growth cones of the cultured cells. This suggests that coactosin might influence axonal growth through its effects on the molecular machinery of actin filament assembly in the growth cone. To verify this possibility, we examined whether coactosin functions in the signaling pathway that regulates actin dynamics in cultured oculomotor axons. During oculomotor nerve formation, oculomotor neurons start to extend their axons from stage 15 both *in vivo* and in primary cell culture (Figure 6A; Landmesser and Pilar, 1972; Hou et al., 2009). As expected, shRNA-mediated knockdown of coactosin decreased the length of cultured oculomotor axons when compared with that of control axons (Figures 6A,B,F). Similarly, axon extension was significantly inhibited with the expression of coactosin K75A (Figures 6C,F). These results indicate that actin-coactosin association is essential for axonal growth.

We further assessed whether coactosin is involved in Rho GTPase signaling which has an epistatic effect on the molecular machinery of actin filament assembly (Sit and Manser, 2011). Expressing a dominant-negative form of RhoA (RhoA-DN) is known to activate actin dynamics and result in axons that are highly motile in both directions (extension and retraction) (Van Aelst and Cline, 2004). Consistent with these findings, we found both long-extended and short-retracted axons on oculomotor neurons with a dominant-negative form of RhoA (Figures 6D,F). The co-transfection of a dominant-negative form of RhoA with coactosin shRNA resulted in axons with a length similar to that of the controls (Figures 6E,F). Given that inhibition of RhoA signaling enhances cofilin activity and actin dynamics, these findings suggest that inhibition of RhoA might compensate for the effects of coactosin knockdown on inactivation of cofilin and actin dynamics.

### Coactosin Was Linked to LIMK1-Mediated Axon Dynamics via Cofilin

**FIGURE 7:**
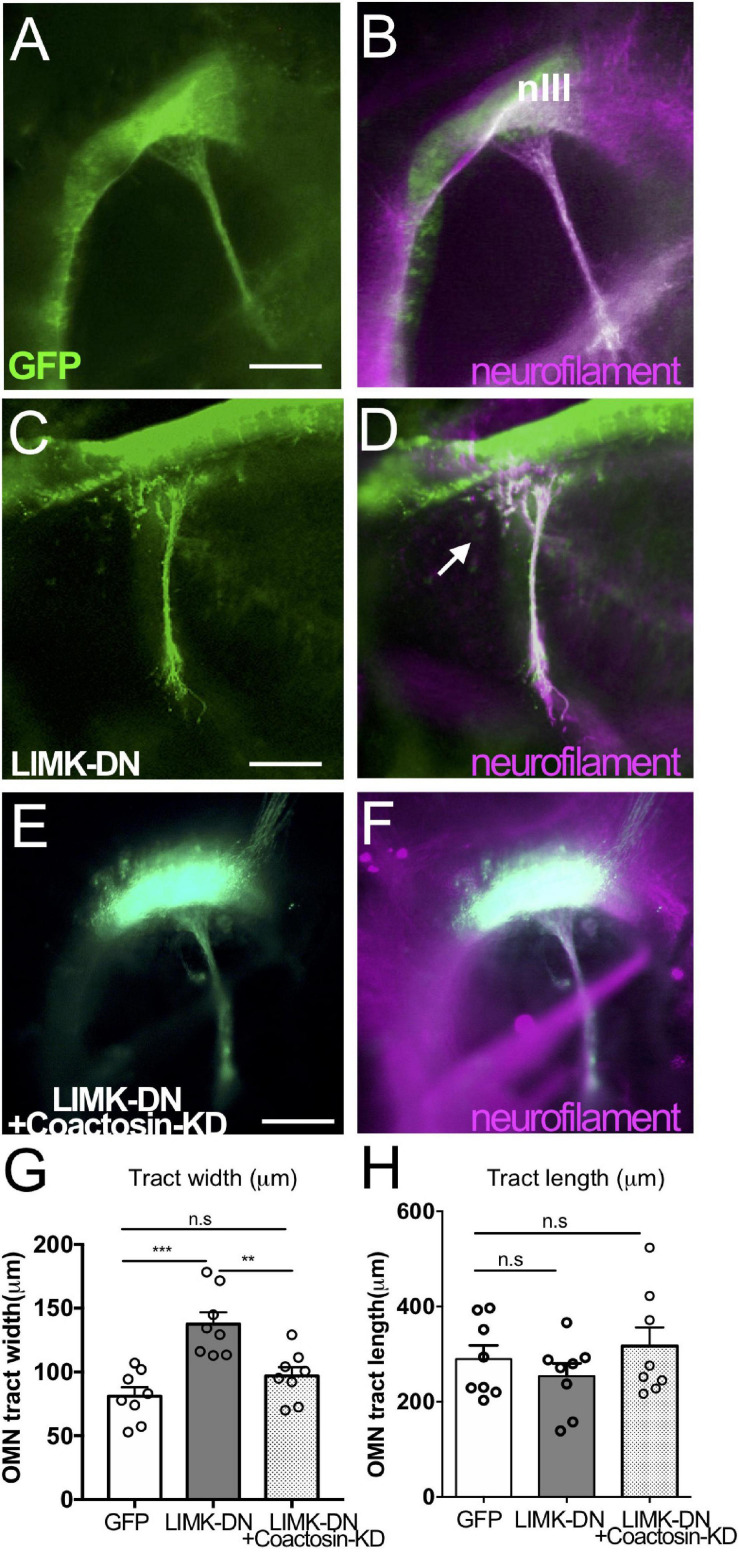
Coactosin function during axon growth under LIMK signals. **(A–F)** Effects of coactosin on the oculomotor nerve of chick embryos. Oculomotor nuclei were transfected with GFP (*green*) **(A,B)**, a dominant-negative form of LIMK1 (LIMK-DN, *green*) **(C,D)**, or LIMK-DN plus coactosin shRNA (coactosin-KD, *green*) **(E,F)**. Double staining of neurofilament (*magenta*) showed an elongation of transfected or untransfected oculomotor nerve. In the control condition, oculomotor axons fasciculate, exit the midbrain, and elongate ventrally. Oculomotor axons defasciculated with LIMK-DN treatment (arrows); however, co-electroporation of LIMK-DN and coactosin shRNA reversed this effect. **(G)** Width of the oculomotor nerve at the exit point from the midbrain. In the LIMK1 inhibition, the defasciculation expanded the width of the nerve (eight embryos for each, **p = 0.0024, ***p = 0.0001, one-way ANOVA) **(H)** Length of oculomotor axons from its nucleus. Note that the effect of coactosin knockdown was rescued by LIMK1 inhibition (eight embryos for each, *p* > 0.05, one-way ANOVA). nIII, oculomotor nucleus, Scale bar: 100 μm.

As well as the regulation of actin dynamics, Rho GTPases also perform numerous other functions in both living cells and developing embryos. Thus, we examined the effect of expressing a dominant-negative LIMK1 mutant in the presence or absence of coactosin in chick embryos (Figure 7). LIMK1, which is a major downstream effector of Rho GTPases in the nervous system, directly regulates cofilin activity via its phosphorylation (Maekawa et al., 1999; Kuhn et al., 2000; Endo et al., 2003), and LIMK1 inhibition upregulates cofilin activity and facilitates actin dynamics, as well as Rho GTPase inhibition (Aizawa et al., 2001; Hsieh et al., 2006). In control embryos, the oculomotor nerve exited the brain and elongated toward the eyeball at stage 17 (Figures 7A,B). In contrast, the axonal nerve was widely defasciculated near the exit point, but its length was not affected when the dominant-negative form of LIMK1 (LIMK-DN) was expressed (Figures 7C,D,G,H). The defasciculation effect of LIMK1 inhibition was abolished by coactosin knockdown (Figures 7E–G). Strikingly, LIMK1 inhibition also rescued the effect of coactosin knockdown, whereby the oculomotor nerve was shorter than the control (see Figure 2), as evidenced by the fact that oculomotor nerve elongation with co-transfection (LIMK-DN + coactosin-KD) was similar to that of the control (Figure 7H; LIMK-DN + coactosin-KD vs. coactosin-KD, eight embryos for each, *p* = 0.0485, one-way ANOVA). This suggests that coactosin knockdown impaired actin dynamics *via* LIMK1-mediated cofilin phosphorylation (inactivation), and thereby restoring cofilin activity under LIMK1 inhibition was sufficient to recover actin dynamics and axonal growth, even with coactosin knockdown. An actin-coactosin association might preserve actin filaments by optimizing cofilin activity in the growth cone.

## Discussion

In this study, we found that coactosin was required for axonal outgrowth both *in vivo* and *in vitro*. SIM analysis showed that coactosin protein accumulated along actin bundles in the growth cone *via* its ADF-H motif and localized closer to the leading edge compared with cofilin. Coactosin knockdown disrupted actin filament arrays and, conversely, overexpression promoted the expansion of lamellipodia and extension of growth cones. In addition, we found that the actin-coactosin association was linked to cofilin activity for the motility of growth cones. Taken together, our findings indicate that coactosin plays an essential role in actin polymerization during axonal outgrowth.

Although the precise molecular mechanisms involved in generating motive force in the growth cone remain unclear, many actin-binding proteins, such as all of the ADF-H family members, including coactosin and cofilin, have been proposed as putative functional molecules in the P-domain of growth cones (Nozumi et al., 2009; Igarashi, 2014, 2019). Indeed, coactosin, an ADF-H-containing protein, is strongly expressed in the nervous system, and primarily localized to highly motile structures as well as other ADF-H family members (Hou et al., 2009, 2013). The action of coactosin in the growth cone in this study resembled its previously reported effects on cellular protrusions in N1E-115 cells and neural crest cells (Hou et al., 2013). Exogenously applied coactosin associated with actin bundles in filopodia and promoted axon and neurite growth, while coactosin knockdown suppressed axon and neurite extension by disrupting actin filaments. Considering its functions shown in *Dictyostelium discoideum* (de Hostos et al., 1993), human myeloid (Provost et al., 2001; Esser et al., 2009), and chick neural crest (Hou et al., 2009, 2013), coactosin is thought to serve as a common factor in actin dynamics (Hellman et al., 2004).

In this study, neither gain nor loss of coactosin functions induced misrouting of the oculomotor nerve; in contrast, LIMK1 inhibition led to defasciculation of this nerve. Many studies have indicated that Rho/LIMK1 inhibition increases growth cone motility by enhancing cofilin activity, thereby disorienting axon growth through repeated retraction and extension events (Aizawa et al., 2001; Hsieh et al., 2006). Coactosin knockdown could cancel the effect of Rho/LIMK1 inhibition on disorienting axons, perhaps by the inactivation of excess cofilin activity. Further investigation of the local function of ADF-H proteins may help to reveal the molecular mechanisms underlying growth cone responses to guidance cues.

Previously, because only confocal microscopy (and not superresolution microscopy) was available, it was difficult to observe the precise and quantified localization of actin-binding proteins, such as coactosin, at the leading edge of growth cones. Around there, F-actin bundles are too densely distributed to recognize using conventional optical microscopy. Application of superresolution microscopy not only allows for the observation of the absolute small-sized samples such as synaptic vesicles (Kittel et al., 2006) but also that of densely distributed ones such as F-actin (Nozumi et al., 2017) and three-dimensionally distributed samples (Zhou et al., 2019).

Here, we visualized the distribution of coactosin in the growth cone using SIM, and its dynamics correlated with that of F-actin (Supplementary Movies 1–3). We first found the precise localization of coactosin, its mutant, cofilin, and *p*-cofilin, and then quantified relationships among them. Coacosin and cofilin had a distinct localization from each other as members of the ADF-H family, even though they biochemically resemble to each other, which suggests that they play different roles for F-actin regulation in axon growth.

The ADF-H domain of coactosin exhibits 25–28% sequence homology to other ADF-H family members, such as cofilin, Abp1, and drebrin (Hou et al., 2009). Importantly, the actin binding site within the ADF-H domain is conserved between coactosin (lys75 in SKYSK) and cofilin (lys95 in SKKED). Thereby, binding to F-actin, and not to G-actin, is considered to be competitive between coactosin and cofilin. In a 3D solution structure, cofilin binds not only monomeric actin but also F-actin on a one-to-one basis. Cofilin has F-actin severing activity by preferentially binding to ADP-actin near the pointed ends of filaments (Yang et al., 1998; Bamburg, 1999), and also limits filament length by binding the barded end via cooperation with capping protein, which depolymerizes the actin filaments (Pollard and Borisy, 2003). In contrast, coactosin is bound to F-actin rather than to monomeric actin (Provost et al., 2001); namely, it is bound to the barbed ends immediately after incorporation of ATP-actin into the filament of F-actin and directly antagonizes the capping protein (Röhrig et al., 1995; Hou et al., 2013). Given the different binding mode of cofilin and coactosin to F-actin, their distinct localization in growth cones might be due to their preference for actin subunits such as ADP-, ADP-Pi- and ATP-actin in filaments rather than competition.

In this study, coactosin association with actin was observed at the leading edge of growth cones, particularly at the tip of filopodia, and hence, gain and loss of function showed coactosin participation in actin polymerization. The tip of filopodia is recognized as the barbed ends rather than the pointed ends of filaments during growth cone extension, and this is where coactosin, but not cofilin, is enriched. The fact that coactosin knockdown led to increased levels of phosphorylated cofilin, accompanied by a disruption in actin filaments, indicated that coactosin acts to preserve the barbed ends as actin polymerization sites. In the absence of coactosin, cofilin may depolymerize filaments around the barbed ends, perhaps cooperating with the capping protein, which is directly antagonized by coactosin (Hou et al., 2013). Alternatively, the absence of coactosin might result in the overproduction of phosphorylated cofilin in homeostatic regulation; phosphorylated cofilin can stop actin polymerization immediately by trapping ADP-actin monomers (Pollard and Borisy, 2003). Regardless, we found that the increased level of LIMK1-phosphorylated cofilin was a major reason for impairment of actin dynamics and axon elongations in coactosin knockdown. Importantly, coactosin is linked to cofilin activity (and hence actin polymerization) through its association with F-actin, but is not likely to be a direct downstream factor of Rho/LIMK signaling because no phosphorylation site of eukaryotic coactosin has been reported (Hasan et al., 2020).

Given that both overexpression and knockdown of cofilin reduce growth cone motility, optimizing cofilin activity is important for the regulation of actin dynamics at the leading edge (Ghosh, 2004; Vitriol and Zheng, 2012). Rho/LIMK1 signaling and/or other accessory proteins involved in actin network organization may form a feedback control loop to restore actin filament stability and, consequently, also cellular integrity (Pollard et al., 2000; Pollard and Borisy, 2003; Chhabra and Higgs, 2007). The homeostatic activity of coactosin and cofilin, the distinct binding to actin filament, and the diverse roles of ADF-H proteins in actin polymerization/depolymerization together contribute to the optimal assembly and disassembly of actin filaments to generate cellular motility.

## Data Availability Statement

The original contributions presented in the study are included in the article/Supplementary Material, further inquiries can be directed to the corresponding authors.

## Ethics Statement

The animal study was reviewed and approved by The Committee for Animal Care at Niigata University.

## Author Contributions

XH, MN, MI, and SS conceptualized and designed the study. XH and SS performed in ovo electroporation and wrote the manuscript. MN, XH, and SS analyzed the observations using SIM. HN provided critical ideas and comments regarding the research. All authors contributed to the article and approved the submitted version.

## Conflict of Interest

The authors declare that the research was conducted in the absence of any commercial or financial relationships that could be construed as a potential conflict of interest.

## Footnotes

1 https://imagej.nih.gov/ij/docs/guide/146-30.html

## Supplementary Material

Supplementary Movie 1 Time-lapse SIM image demonstrating that coactosin is localized to the actin bundles. EGFP-coactosin (*green*) and mCherry-actin (*red*) were co-expressed in NG108-15 cells. Coactosin accumulated around the barbed ends of filaments shortly after incorporation of ATP-actin. Corresponding to Figure 4.

Supplementary Movie 2 Time-lapse SIM image when EGFP and mCherry-actin (*white*) were co-expressed in NG108-15 cells. A point at the leading edge of the growth cone was tracked using MTrackJ. Corresponding to Figure 4.

Supplementary Movie 3 Time-lapse SIM image demonstrating a rapid expansion of lamellipodia and an accelerated extension of the growth cone when EGFP-coactosin and mCherry-actin (*white*) were co-expressed in NG108-15 cells. A point at the leading edge of the growth cone was tracked using MTrackJ. Corresponding to Figure 4.
